# Asymptomatic Salmonella Myocarditis: A Case Report of a Rare Entity

**DOI:** 10.7759/cureus.54502

**Published:** 2024-02-19

**Authors:** Rizwan Ullah, Suleman Khan, Aftab Ahmad, Syed Owais Haseeb, Saad Karim

**Affiliations:** 1 Internal Medicine, Hayatabad Medical Complex Peshawar, Peshawar, PAK; 2 Geriatrics, Cork University Hospital, Cork, IRL

**Keywords:** rare case report, asymptomatic myocarditis, clinician awareness, cardiac mri, cardiovascular complications, enteric fever

## Abstract

Enteric fever typically displays symptoms like high fever, abdominal pain, constipation, and headaches, primarily affecting the digestive system. While it is commonly seen as a gastrointestinal infection, it can also lead to rare but significant cardiovascular issues. There have been only a few reported cases of enteric fever causing heart manifestations. We present a case of a young male with enteric fever-induced myocarditis, which, due to its rarity, can be challenging to diagnose and is essentially a diagnosis of exclusion. Cardiac MRI (CMR) is crucial for diagnosis, supported by ECG, echocardiograms, and troponin levels. The treatment involves standard approaches for cardiomyopathy, including angiotensin-converting enzyme (ACE) inhibitors, beta-blockers, and diuretics. However, our patient presented as a case of asymptomatic myocarditis and fully recovered with treatment without any long-lasting heart problems. Our study aims to contribute to the limited body of knowledge on heart-related complications of enteric fever, raising awareness among clinicians of such presentations in enteric fever cases.

## Introduction

Enteric fever, which is caused by the bacteria Salmonella typhi, is a major source of disease burden in developing and underdeveloped nations. The clinical manifestation of enteric fever can range from a low-grade fever with symptoms including headache, fatigue, malaise, loss of appetite, cough, constipation, and rose spots to more severe cases that lead to potentially fatal complications like encephalitis, cranial neuritis, intestinal perforations, and, infrequently, myocarditis [1]. When a high index of clinical suspicion is not maintained for myocarditis, it might be readily overlooked as its clinical signs are usually nonspecific. In immunocompetent people, myocarditis is rarely caused by bacteria [1]. Instead, it is typically caused by viruses such as the Coxsackievirus or enteroviruses. Although Salmonella typically results in enteric fever, it can also, in rare cases, cause myocarditis in healthy people [2].

Patients with enteric myocarditis exhibit symptoms identical to other myocarditis cases. They will experience hypoxemia, chest discomfort, and dyspnea. There will be signs and symptoms of heart failure as a result of the inflammation that follows, which will worsen the heart's systolic function [3]. However, it has been found that 1-5% of typhoid fever patients had toxic myocarditis [3]. A study conducted in the medical department of KMC, Mangalore, India, examined 100 cases of enteric fever that were proven to be bacterial or serological. Of note, 46 patients had myocarditis detected on the ECG, and seven cases showed clinical evidence of the condition. Myocarditis risk was greatest in those with systemic involvement (p<0.01). Therefore, it is widely accepted that an ECG must be performed in every episode of enteric fever. Patients with alterations in their ECG should also be closely monitored for clinical signs of myocarditis [4].

Myocarditis can be diagnosed using several novel diagnostic techniques, including cardiac MRI (CMR). Other available tests include the Echo (echocardiogram) and ECG, which serve as auxiliary diagnostic tools with often nonspecific results. Patients with life-threatening arrhythmia, acute dilated cardiomyopathy linked to hemodynamic compromise, or those whose condition does not improve with standard supportive care, may benefit from an endomyocardial biopsy. The diagnosis of myocarditis is predicated on excluding the more prevalent conditions, such as viral infections [3].

The primary course of treatment for enteric myocarditis involves antibiotics, based on the sensitivity and culture of the local antibiotic population, as well as conventional heart failure drugs such as beta-blockers, angiotensin-converting enzyme (ACE) inhibitors, angiotensin receptor blockers (ARBs), and diuretics [5]. Although the results of enteric-induced myocarditis vary from case to case, most patients get their normal cardiac function restored, and the prognosis is usually good [6].

## Case presentation

A 22-year-old male with no previous comorbidities presented to us with high-grade fever associated with chills and rigors, nonspecific chest pain, and occasional dry cough for seven days. He had taken multiple antibiotics (Tab. cefixime 400 mg OD for six days, injection artesunate 120 mg OD for four days) without any response. His fever was peaking up to 103/104 °F. On examination, he had a fever of 103.8 °F with a pulse rate of 93 bpm, a blood pressure of 110/70 mmHg, and a toxic appearance. His abdomen was soft and non-tender with no visceromegaly. His chest auscultation showed normal vesicular breath sounds with no evidence of any respiratory infection. His cardiovascular examination did not reveal any significant findings. The patient had no edema. His lab reports are presented in Table 1.

**Table 1:** All laboratory investigations during hospital stay ESR: erythrocyte sedimentation rate; HDL: high-density lipoprotein; LDL: low-density lipoprotein

Labs	Results on day 1	Result on day 7	Reference range
White blood cells (cells/mm^3^)	9700	8300	4000-11000
Hemoglobin (g/dl)	16	15	13-17
Platelets (cells/mm^3^)	226,000	284,000	150000-400000
Troponin (ng/l)	750	28	0-33
LDH (U/L)	857	35	0-33
ESR (mm/hr)	41	14	0-15
Cholesterol (mg/dl)	165	157	<200
HDL (mg/dl)	30	31	35-60
LDL (mg/dl)	115	108	<130
Triglycerides (mg/dl)	89	93	<150

**Figure 1:**
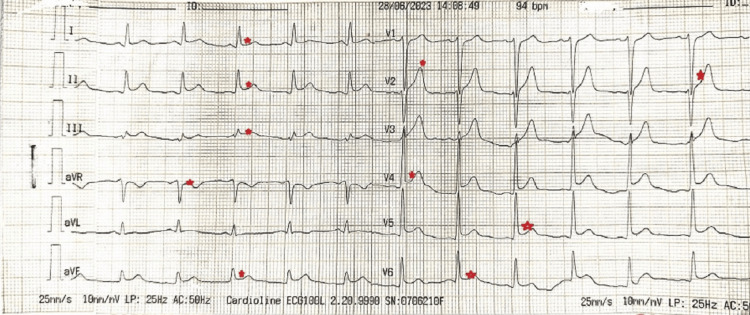
Electrocardiogram on arrival showing diffused ST segment and T-wave changes (red stars)

The abdominal ultrasound yielded normal results, while an ECG conducted for chest pain revealed nonspecific ST elevations and T-wave changes, as illustrated in Figure 1. Subsequent Trop I testing indicated significantly elevated levels. Although two previous Echo tests conducted three days earlier had appeared normal, a recent Echo revealed regional wall abnormalities with fair left ventricular function and normal ventricular sizes. Serology tests for dengue, Coxsackie virus, HIV, Epstein-Barr virus, and HSV were negative. Malarial parasites were not seen. Chest X-ray and high-resolution CT (HRCT) also presented normal findings.

**Figure 2:**
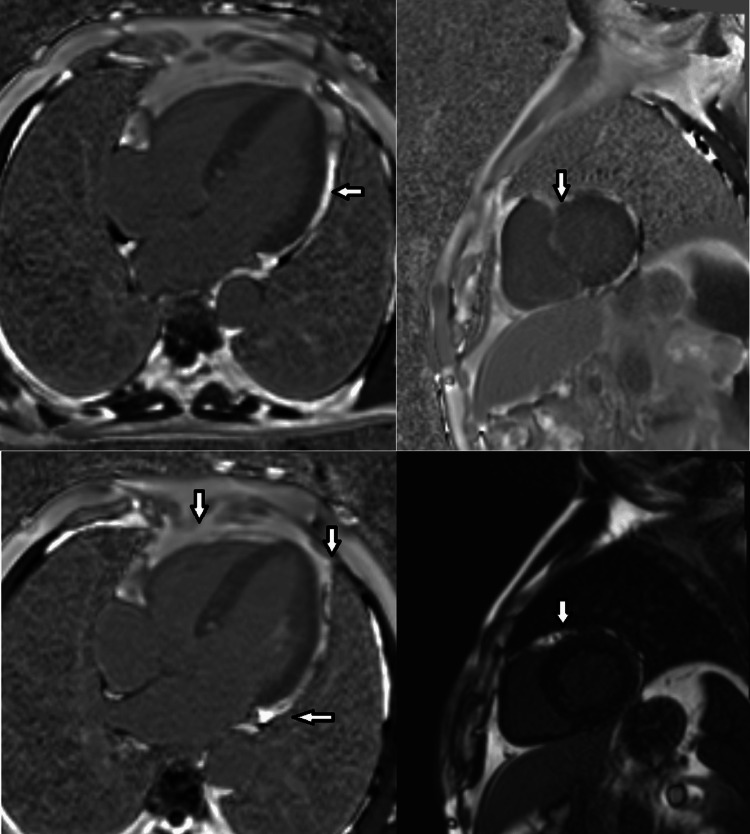
CMR showing increased wall thickness, multiple regions displaying heightened T2 signal intensity (regional edema), and increased ratio of early gadolinium enhancement in the myocardium (white arrows) CMR: cardiac magnetic resonance imaging

Given the patient's history of multiple antibiotic use (no proper record was available), empirical therapy was initiated with Inj. azithromycin 500 mg OD (once a day) and Inj. meropenem 1 gm TDS (thrice a day), along with supportive treatment (dexamethasone, diuretics, vitamin-B complex). Dual antibiotics were chosen based on local guidelines due to increasing resistance to enteric fever in the domestic setup. Blood culture results confirmed the presence of Salmonella typhi, sensitive to azithromycin and meropenem, but resistant to ceftriaxone, ampicillin, chloramphenicol, and ciprofloxacin. Based on a high suspicion of enteric myocarditis, a CMR was scheduled and revealed augmented wall thickness, sporadic regions displaying heightened T2 signal intensity suggestive of localized or regional edema, and an elevated ratio of early gadolinium enhancement in the myocardium (Figure 2).

**Figure 3:**
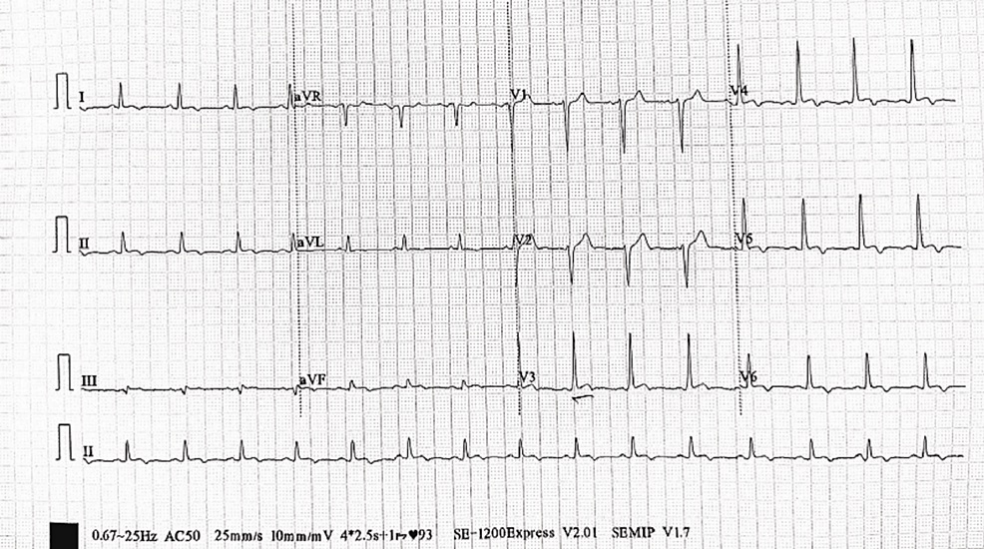
Electrocardiography on discharge showing normal ST wave

The patient responded well to treatment and his lab results improved. Given the prevalence of enteric fever in the region, the patient's response to treatment, imaging findings, and the blood culture report, a diagnosis of enteric fever complicated by myocarditis was established. On the seventh day, repeat assessments including Echo, ECG (Figure 3), and Trop I returned to normal, and the patient was discharged in a completely healthy condition.

## Discussion

Enteric fever is a condition that affects multiple organ systems. Intestinal perforation is the most frequent consequence, which often arises in the third week of the disease. Hepatobiliary, central nervous system, pulmonary, and rheumatological issues are among the other organ system complications observed [7]. A range of cardiovascular problems, including myocarditis and endocarditis as the primary consequences, and pericarditis and arteritis, which occur less frequently, were observed in 1-5% of patients. Myocarditis might resemble ischemia in younger patients in terms of its clinical and electrocardiographic characteristics. Localized inflammation or coronary spasm may be the cause of any apparent ischemia linked to myocarditis. When other potential causes of elevated cardiac enzymes and electrocardiographic abnormalities are ruled out, a diagnosis of myocarditis is frequently made. CMR may show myocardial edema and myocyte degeneration [8].

Myocarditis often presents without any noticeable symptoms, encompassing a range of clinical manifestations from mild local inflammation to severe congestive heart failure. The spectrum includes atypical chest pain, along with potential indicators like dizziness, syncope, and palpitations arising from atrial and ventricular arrhythmias [4]. The lack of specificity in symptoms makes them easy to overlook in the absence of a high degree of clinical suspicion [8]. In our case, the patient displayed atypical chest pain despite maintaining normal cardiac hemodynamics. The ECG revealed nonspecific ST changes and T-wave alterations. Cardiac enzymes were elevated. Echocardiography exhibited regional wall abnormalities, while both left and right ventricular functions were fair, with normal ventricular sizes and an absence of pericardial fluids. Notably, despite being clinically asymptomatic and having normal cardiac hemodynamics, the blood culture sensitivity results, the patient's response to treatment, and CMR findings were strongly suggestive of myocarditis.

Myocarditis originating from enteric sources can result in cardiovascular insufficiency, a prevalent cause of mortality during the second week of illness. The development of Salmonella typhi is believed to be influenced by factors such as the size of the initial inoculum, virulence, the response of the immune host, prior exposure history, and local protective elements [5].

The treatment for concomitant cardiomyopathy and enteric myocarditis is similar to that for other forms of myocarditis. It is advisable to start treating enteric myocarditis with supportive care, which is effective; some studies also suggest that dexamethasone is helpful. It is preferable to stay away from non-steroidal inflammatory drugs (NSAIDs), to avoid interfering with the myocardium's repair process. The neurohormonal loop that causes systolic dysfunction can be blocked by starting beta-blockers, diuretics, and ACE inhibitors [5]. Our patient was started on azithromycin 500 mg OD and meropenem 1 gm TDS according to our local sensitivity patterns. We also promptly started supportive treatment including dexamethasone, diuretics, and vitamin complex. The patient responded well and was subsequently discharged home in a healthy condition.

## Conclusions

Enteric fever is associated with various manifestations; it can be asymptomatic or fatal, and the identification of enteric myocarditis is a rare yet crucial diagnosis that demands timely intervention. Due to the rarity of myocarditis in cases of enteric fever, there is a risk of oversight, emphasizing the need for maintaining a high level of suspicion. Supportive evidence for diagnosing enteric myocarditis can be obtained through troponin levels and echocardiographic findings, with confirmation provided by CMR. Swift initiation of treatment is paramount, as it can facilitate rapid recovery and prevent permanent myocardial damage. The core of therapy involves diuretics, ACE inhibitors or ARBs, beta-blockers, and appropriate antibiotics guided by culture and sensitivity reports. Continuous monitoring of ventricular function through serial echocardiograms is essential until normal function is restored. Our case was unique in that the patient had asymptomatic myocarditis that showed rapid recovery with antibiotics and supportive treatment.
